# Vorwort der Herausgeber

**DOI:** 10.1007/s11943-022-00304-8

**Published:** 2022-03-16

**Authors:** Timo Schmid, Markus Zwick

**Affiliations:** Lehrstuhl für Statistik und Ökonometrie, Otto-Friedrich-Universität Bamberg, Feldkirchenstraße 21, 96045 Bamberg, Deutschland

Liebe Leser und Leserinnen,

mit dieser ersten Ausgabe des Jahres 2022 laden wir Sie wieder herzlich ein, unser Journal zu lesen oder dieses durch ihre eigenen Zusendungen zu bereichern. Neben dem Interview mit Bernd Fitzenberger finden sich in dieser Ausgabe interessante Artikel zur Entwicklung der Grundschulversorgung sowie einen methodischen Beitrag zur Darstellung statistischer Daten auf Karten mittels Dichten am Beispiel Berliner Wahldaten. Wie die meisten Lebensbereiche wird auch das praktische und methodische statistische Arbeiten weiterhin von der globalen COVID-19-Pandemie beeinflusst. Zu diesem Thema liefern die ersten beiden Artikel dieser Ausgabe neue Erkenntnisse.

Einer statistischen Herausforderung der COVID-19-Pandemie stellen sich De Nicola et al. (2022) mit ihrer Schätzung einer verlässlichen Zahl der Todesopfer durch die Pandemie. Zweifelsohne geht die Corona-Pandemie mit einer erhöhten messbaren Zahl an Todesfällen in der Bevölkerung einher. De Nicola et al. (2022) zeigen jedoch, dass die Quantifizierung der Übersterblichkeit keineswegs trivial ist, denn die alleinige Stützung auf öffentlich gemeldete COVID-19-assoziierte Todesfälle birgt die Gefahr statistischer Verzerrungen. Eine Lösung findet sich im Vergleich der beobachteten Gesamtmortalität im Jahr 2020 mit der Zahl der Todesfälle, die im selben Jahr ohne das Auftreten von COVID-19 zu erwarten gewesen wäre. De Nicola et al. (2022) analysieren zwei Methoden zur Berechnung der erwarteten Sterblichkeit auf jährlicher bzw. wöchentlicher Ebene, um Schätzungen für die Übersterblichkeit in Deutschland 2020 zu erhalten. Beide Ansätze führen zu ähnlichen Ergebnissen auf der Gesamtebene und zeigen, dass das Jahr 2020 durch eine allgemeine Übersterblichkeit von etwa 1 % gekennzeichnet ist, wobei starke altersspezifische sowie saisonale Unterschiede hervortreten.

De Nicola und Kauermann (2022) wenden die Methode zur Berechnung der Übersterblichkeit unter Berücksichtigung der Altersstruktur aus De Nicola et al. (2022) für die neusten Daten aus dem Jahr 2021 an. Ihre aktualisierten Berechnungen unterstützen die Robustheit ihrer bisherigen Ergebnisse und führen zur Erkenntnis einer vorläufigen Übersterblichkeit von 2,3 % für das Jahr 2021. Als prägnant erweist sich der Umstand, dass besonders die Todesfälle der Altersgruppe 60–79 Jahre überproportional beitragen.

Erfurth et al. (2021) zeigen in ihrem Beitrag eine neue Möglichkeit, wie Karten für räumliche Daten auf der Ebene administrativer Flächeneinheiten auf Basis von Dichten dargestellt werden können. Die Standarddarstellung statistischer Daten geht oft von einer Gleichverteilung dieser innerhalb einer (administrativen) geographischen Referenzeinheit aus. Diese Annahme bringt mehrere Nachteile wie z. B. das Erschweren der Identifikation von regionalen Clustern wegen Sprüngen an den Grenzen der regionalen Einheiten mit sich. Erfurth et al. (2021) modifizieren den simulierten EM (SEM) Algorithmus von Celeux et al. (1996) um in einem zweistufigen Prozess erst auf Basis einer Dichteschätzung mit den Aggregatsinformationen konsistente Beobachtungen zu simulieren, um dann in einem zweiten Schritt einen Kerndichteschätzer auf die simulierten Daten anzuwenden, der die nächste Dichteschätzung liefert. Die Autoren evaluieren ihre auf diesem Verfahren basierenden Karten mit verschiedenen Standardkarten auf Basis eines lokalen Wählerregisters mit bekannten Adressen. Die Methode wird dann angewendet, um Karten zu generieren, die die Berliner Ergebnisse zur Wahl des Abgeordnetenhauses 2016 darstellen. Der Vergleich dieser Karten mit Standard-Choroplethenkarten illustriert die neuen Möglichkeiten der von den Autoren entwickelten Methode.

In ihrem Artikel „Eine kleinräumige Mikrosimulationsstudie am Beispiel der Stadt Trier“ schätzen Dräger et al. (2022) die zukünftige Entwicklung der Grundschulversorgung der Stadt Trier mit einem Mikrosimulationsmodell. Das Modell erlaubt dabei auch die Miteinbeziehung ausgewählter Wanderungsszenarien. Primäres Ziel ist es, die Anzahl an Schülerinnen und Schülern in Grundschulen und auch deren Verteilung auf die Klassenstufen zu prognostizieren. Dafür werden die Aktionen und Interaktionen von Individuen, die auf einer realitätsnahen synthetischen Basispopulation basieren, mit Hilfe von verschiedenen Modulen die z. B. Geburten, Tode, Wanderungen oder den Beziehungsstatus simuliert. Da die Basispopulation auf Daten für das Jahr 2011 basiert und Daten der realen Entwicklung bis 2018 vorliegen, können die vom Mikrosimulationsmodell prognostizierten Daten mit den tatsächlichen verglichen werden. Unter der Berücksichtigung nicht vorhersehbarer externer Schocks, wie den Migrationsbewegungen um das Jahr 2015, liefert es eine gute Vergleichbarkeit mit den tatsächlichen Daten. Schließlich wird das Modell genutzt, um die Grundschülerzahlen unter verschiedenen Szenarien bis ins Jahr 2061 zu prognostizieren.

Im Interview dieser Ausgabe (vgl. Krämer, 2022) gewährt Bernd Fitzenberger im Gespräch mit Walter Krämer facettenreiche Einblicke unter anderem in die Themenfelder der universitären Lehre und Entwicklungen des deutschen Arbeitsmarkts. Bernd Fitzenberger hat in Konstanz Volkswirtschaftslehre und Mathematik studiert, um im Anschluss an der renommierten Universität Stanford in Economics zu promovieren. Nach verschiedenen Professuren an mehreren deutschen Universitäten ist er nun der Direktor des Instituts für Arbeitsmarkt- und Berufsforschung (IAB), verbunden mit einer Professur für Quantitative Arbeitsökonomik an der Friedrich-Alexander-Universität Erlangen-Nürnberg. Neben anderen Themen spricht der empirische Arbeitsmarktforscher im Interview über Verbesserungspotentiale an deutschen Universitäten im Vergleich zu amerikanischen Universitäten und über Herausforderungen in der Lehre und Forschung im Fach Statistik, die aus der Verstreuung des Fachs auf verschiedenen Disziplinen resultieren. Weiterhin berühren die Interviewpartner auch das politisch gewichtige Thema der benötigten Zuwanderung, um einem Arbeitskräftemangel am deutschen Arbeitsmarkt entgegenzuwirken.

Mit dem Jahreswechsel hat sich auch der Herausgeberbeirat ein wenig gewandelt. Für den Herausgeberbeirat konnten wir Katharina Schüller, Andreas Kladroba, Philipp Otto, Marcel Preising und Tobias Schoch neu gewinnen. Ihnen der Dank für ihre Bereitschaft künftig gemeinsam mit den weiteren Beiratsmitgliedern und uns die Geschicke der Zeitschrift zu lenken.

Nun wünschen wir Ihnen, liebe Leserinnen und Leser, viel Spaß bei der Lektüre der ersten Ausgabe des AStA Wirtschafts- und Sozialstatistisches Archivs in diesem Jahr.

Timo Schmid und Markus Zwick
